# How can common errors in left heart echocardiography be avoided? An expert proposalby the German Working Group of Cardiovascular Ultrasound

**DOI:** 10.1007/s00392-025-02780-8

**Published:** 2025-10-27

**Authors:** Andreas Hagendorff, Stephan Stöbe, Andreas Helfen, Fabian Knebel, Ertunc Altiok, Wolfgang Fehske, Tarek Bekfani, Ole-A. Breithardt, Aydan Ewers, Ali Hamadanchi, Henrik ten Freyhaus, Dariush Haghi, Jan Knierim, Sebastian Kruck, Karsten Lenk, Joscha Kandels, Nicolas Merke, Raluca-Ileana Mincu, Elena Romero Dorta, Tobias Ruf, Christoph Sinning, Jens-Gerrit Kluge, Roland Brandt, Maximilian von Roeder, Jens-Uwe Voigt, Sebastian Ewen, Bernhard Haring

**Affiliations:** 1 Department of Cardiology, University Hospital Leipzig AöR, Leipzig, Germany; 2 Medizinische Klinik II - Kardiologie, Krankenhaus St. Elisabeth Halle, Halle, Germany; 3 Department of Cardiology, Katholische St. Paulus Gesellschaft, St.-Marien-Hospital Lünen, Lünen, Germany; 4 Department of Internal Medicine II, Sana Klinikum Lichtenberg, CardiologyBerlin, Germany; 5 Department of Cardiology, Angiology, and Intensive Medicine, University Hospital Aachen, Aachen, Germany; 6 Kardiologische Praxis Köln, Cologne, Germany; 7 Department of Cardiology and Angiology, University Hospital Magdeburg AöR, Magdeburg, Germany; 8 Kardiologische Privatpraxis Kassel, Kassel, Germany; 9 Department of Cardiology and Angiology, BG University Hospital Bergmannsheil Bochum, Bochum, Germany; 10 Department of Cardiology, University of Jena, Jena, Germany; 11 Department of Internal Medicine III, Cardiology, University of Cologne, Cologne, Germany; 12 Kardiologische Praxisklinik Ludwigshafen-Akademische Lehrpraxis of the University of Heidelberg, Ludwigshafen, Germany; 13 Department of Internal Medicine and Cardiology, Berlin, Germany; 14 Praxis Für Kardiologie Cardio Centrum Ludwigsburg, Ludwigsburg, Germany; 15 Department of Cardiothoracic and Vascular Surgery, Deutsches Herzzentrum Charité Berlin, Berlin, Germany; 16 Department of Cardiology and Angiology, University of Essen, Essen, Germany; 17 Department of Cardiology, Angiology and Intensive Care Medicine, University of Berlin, Deutsches Herzzentrum Charité Berlin, Campus Mitte, Berlin, Germany; 18 Department of Cardiology, Center of Cardiology, Heart Valve Center, University Medical Center Mainz, University of Mainz, Mainz, Germany; 19 Department of Cardiology, University Heart and Vascular Center Hamburg, German Centre of Cardiovascular Research (DZHK), Hamburg, Germany; 20 Abteilung Kardiologie Und Internistische Intensivmedizin, Ev. Krankenhaus Göttingen-Weende, Göttingen, Germany; 21 Department of Cardiology, Kerckhoff Klinik GmbH, Bad Nauheim, Germany; 22 Department of Cardiology, Heart Center Leipzig, Leipzig, Germany; 23 Department of Cardiovascular Diseases, Department of Cardiovascular Sciences, University Hospital Gasthuisberg, and, Catholic University Leuven , Louvain, Belgium; 24 Department of Cardiology and Intensive Care Medicine, Schwarzwald-Baar Klinik, Villingen-Schwenningen, Germany; 25 Department of Cardiology, University Heart Center Freiburg, Bad Krotzingen, Freiburg, Germany; 26 Department of Cardiology, Angiology and Intensive Care Medicine, University Hospital Homburg/Saar, Homburg, Germany; 27 Department of Medicine IV, Vienna Healthcare Group, Clinic Hietzing, Vienna, Austria

**Keywords:** Basic echocardiography, Conventional parameters, Cardiac wall thickness, Cardiac volumes, Strain measurements

## Abstract

Echocardiography is the most commonly used imaging modality in cardiology and serves as a crucial first step in diagnosis and therapeutic decision making. This expert proposal aims to address methodological requirements in order to make the correct diagnosis and prevent diagnoses from being overlooked due to improper use.

**Graphical Abstract:**

**Figure:**
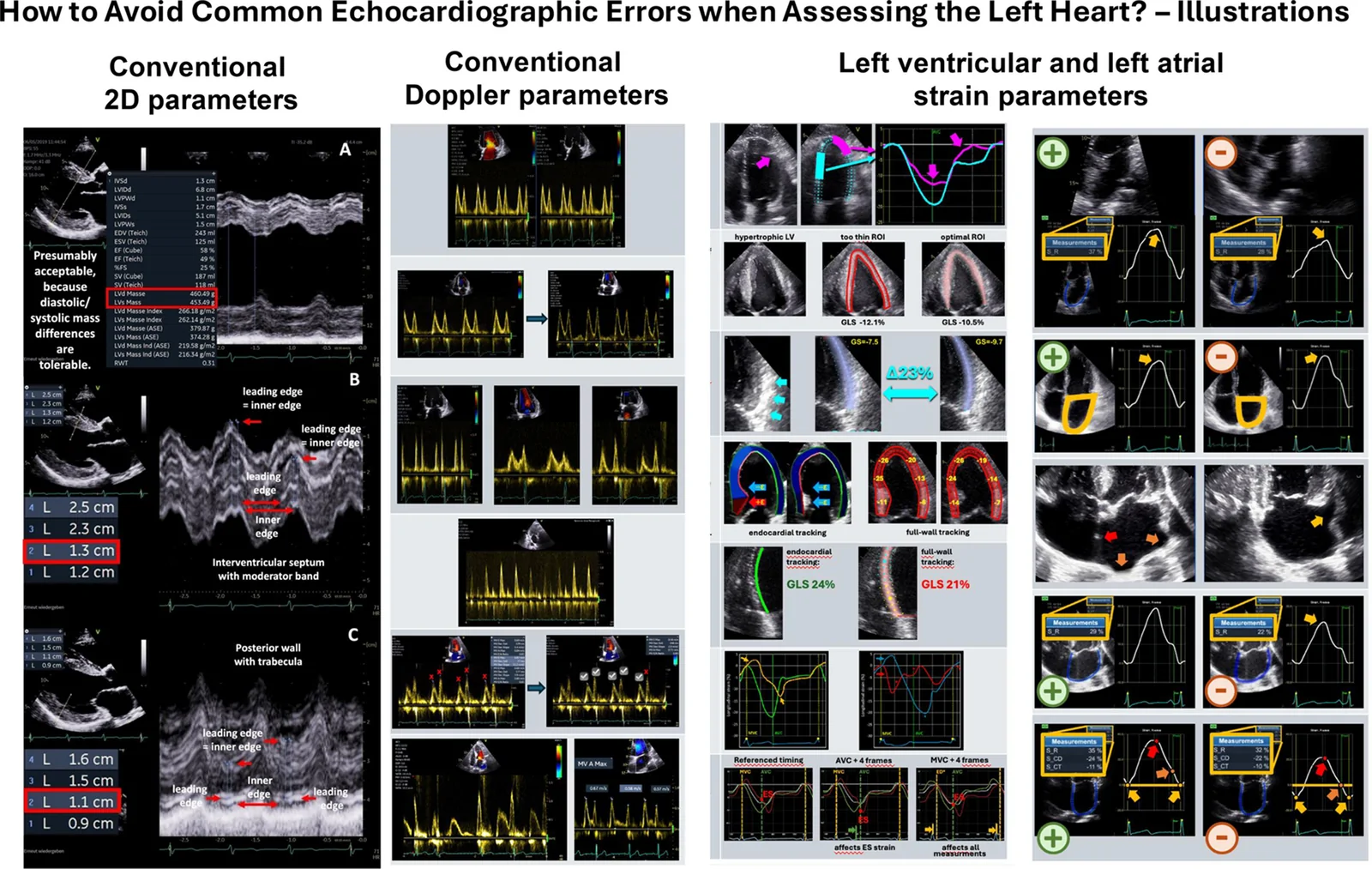

**Supplementary Information:**

The online version contains supplementary material available at https://doi.org/10.1007/s00392-025-02780-8.

## Introduction

In modern echocardiography, reproducible measurement of basic quantitative cardiac parameters is becoming increasingly important to completement other imaging techniques. Standardized image acquisition and documentation is a crucial prerequisite for avoiding errors in all cardiac diseases [1–5]. Precise and reproducible measurements depend on high quality images [6, 7]. Even advanced machine learning algorithms and artificial intelligence (AI) cannot compensate for poor image acquisitions. The following expert proposal discusses the most important sources of error in the determination of basic echocardiographic parameters and provides practical tips on how to avoid them.

### General considerations for optimizing ultrasound settings

The ultrasound settings for all echocardiographic modalities have a significant influence on image quality. Due to varying individual sonographic characteristics, settings should be adjusted individually to achieve optimal image acquisition. Spectral and color Doppler settings are optimized by adjusting the Doppler ultrasound frequency, low velocity reject, velocity scale, and gain. The ultrasound frequency generally determines the spatial resolution, i.e., the higher the frequency, the better the spatial resolution—however, higher frequencies limit penetration depth. In general, lower ultrasound frequencies and higher gain settings lead to an overestimation of the thickness of boundary lines. Images with better spatial resolution and adjusted brightness naturally show dimensions that are comparable to the respective anatomical conditions—especially, no overexposure. The lateral resolution is largely determined by the scan line density and the frame rate itself, with the width of the sector playing an important role in addition to the ultrasound frequency. Color overgaining, pixel smoothing, and reduced low velocity reject leads to color superimposition on the tissue and thus to an overestimation of almost all color Doppler echocardiographic parameters in the assessment of valvular regurgitations. The frame rate of color Doppler ultrasound must be sufficiently high to assess systolic turbulences, taking into account the heart rate, so that at least 6–8 images are recorded during systole.

Key points: Spectral and color Doppler settings should be optimized individually. The main sources of errors with unfavorable ultrasound settings are underestimation of diameters and areas due to too thick, overexposed contours, and underestimation of planimetrically determined valve opening areas due to too low frame rates. Inadequate color settings generally lead to misjudgments of the severity of regurgitations.

### Linear left ventricular and left atrial measurements in 2D echocardiography and M-mode

The echocardiographic assessment of the cardiac cavities in 2D echocardiography is based on length and area measurements and the subsequent calculation of volumes using established geometric models and mathematical equations [8, 9]. Traditionally linear left ventricular (LV) geometry is assessed in M-mode by measuring the LV-diameter, LV wall thickness, LV size. and LV mass [10–12]. LV volumes are derived from measured diameters using standard geometric formulas [13, 14]. LV wall thickness is reported as the thickness of the interventricular septum (IVS) and the posterior LV wall (LVPW), and LV size is reported as the LV end-diastolic and end-systolic diameters (LVEDD, LVESD). All of these values should currently be determined from 2D images in the standardized parasternal long axis (pLAX). However, they can also be assessed in any non-standardized LAX using a so-called anatomical M-mode (Supplementary Fig. S1).

End-diastole is defined as the maximum expansion of the left ventricle after mitral valve closure, while end-systole is defined as the minimum expansion of the left ventricle after aortic valve closure. The determination of end-diastole and end-systole should be primarily based on mechanical expansion, rather than on electrical activity recorded by the ECG, especially in patients with arrhythmias [15]. Linear measurements should be made using 2D images at the junction of the chordae tendineae to the edges of the mitral valve leaflets parallel to the mitral valve annulus to avoid oblique sections that can occur when using the conventional M-mode technique [15, 16].

Oblique views are the main source of error in both 2D and M-Mode images. All measurements should be performed as centrally as possible and perpendicular to the LV longitudinal axis. Oblique pLAX planes tend to overestimate LV wall thickness and underestimate LV diameters. Simultaneous biplane scanning of the pLAX and parasternal short axis (pSAX) with a multidimensional probe can be used to verify central LV alignment. When using the monoplane scanning technique, it is necessary to perform a precise 90° counterclockwise rotation after setting the pSAX without tilting the transducer in any direction to avoid secant intersections of the pLAX. A significant source of error in measuring LV wall thickness in 2D and in M-mode is the inaccurate representation of the posterior (= inferolateral) LV wall due to the limitations of parasternal scanning, which impairs the ability to clearly identify the endocardial and epicardial lines of the posterior wall. These limitations can be circumvented by optimizing image quality and by imaging the heart from a more caudal and/or lateral window for linear measurements in 2D images and anatomical M-mode. Non-standardized short-axis views can be recognized by an oval shaped LV.

Measurement of the thinner IVS near the LV outflow tract (LVOT) should be avoided as this may lead to an underestimation the actual thickness and severity of LV remodeling and LV hypertrophy [15]. The presence of an IVS bulge (also known as a sigmoid septum) is a common finding in the elderly and refers to an isolated thickening of the basal septum, resulting in a sigmoid configuration. In the presence of sigmoid septum, IVS measurements should be performed apical to the bulge [4, 15, 17]. Similarly, LV diameters should also be measured apical to the bulge.

When measuring the IVS, care must be taken to ensure that no parts of the right ventricular (RV) trabeculae are included, as this would lead to an overestimation of the IVS thickness. The presence of a hyperechoic line in the septum often reflects a change in fiber orientation separating the LV from the RV septum [18]. Parts of the mitral valve apparatus must also be excluded from the measurements. Particular attention must be paid to the endocardial border of the posterior wall, as parts of the posterolateral papillary muscle are sometimes visible, but these structures are not part of the LV posterior wall and should instead be included in the LV diameter (Supplementary Fig. S2). Measurements should be performed according to the PENN- “inner edge-inner edge”-convention. This convention means that the lines of the endocardium are excluded from the LV wall thicknesses and included in the assessment of LVEDD [12]. Therefore, the interface between the myocardial wall and the cardiac cavities as well as the interface between the LV outer wall and the pericardium should be marked for measurement (Fig. 2). Compared to measurements of linear parameters in the 2D image, conventional M-mode offers higher spatial and temporal resolution, which often allows better differentiation of the endocardial and epicardial boundaries and anatomical structures—e.g., RV trabeculae, moderator band and muscle fibers of the posteromedial papillary muscle—especially when image quality is poor. Visual tracking of the myocardial speckles in M-mode over time often facilitates the assignment of measurement points to target structures [7, 19]. The spatial resolution of anatomical M-mode decreases with increasing orientation perpendicular to the direction of the ultrasound beam.

M-mode parameters such as LV wall thickness [11], relative wall thickness (RWT) [20, 21], and LV mass (LVM) [12, 22] continue to be used in daily clinical practice. LV remodeling can be described by the LV remodeling index (LVRI) which is defined as the ratio of LV mass to biplane LV end-diastolic volume (LVEDV) [23]. However, 3D echocardiography is superior to 2D examination for assessing LV mass (Supplementary Fig. S1) [24].

Linear left atrial (LA) dimensions can be obtained from any standard view. The most commonly used linear LA dimension is the antero-posterior (AP) diameter, which is determined in the pLAX at end-systole using 2D or M-mode echocardiography [15, 25]. However, linear dimensions are no longer recommended for characterizing LA size.

Key points: Linear 2D measurements of LV parameters should only be performed in documentation where the target structures can be clearly marked. Foreshortening of the central LV long axis can be controlled and excluded by biplane scanning. With anatomical M-mode, measurements can also be performed well from non-standardized long axis views.

### LV- and LA-volume measurements by 2D echocardiography

Biplane LV planimetry using the modified Simpson’s rule has become the standard for determining end-diastolic and end-systolic LV volumes (LVEDV, LVESV) and LV ejection fraction (LVEF) in 2D echocardiography [26, 27]. When using the Simpson’s method for 2D LV planimetry, it is assumed that the left ventricle is an elongated ellipsoid. This method does not take into account abnormal or asymmetrical ventricular shapes.

The distance between mitral annular plane and the apex corresponds to the LV length [15, 28]. The LV length between 4- and 2 chamber view (4-ChV, 2-ChV) should not differ by more than 10% and can also be compared with the LV length in the apical LAX (aLAX) [15].

The patient should be positioned on their left side to bring the heart closer to the chest wall. Sonographic access is further improved placing the patient’s left arm under their head to increase the distance between the ribs and thus create larger windows for the ultrasound beam [29]. Standardized image acquisition is best performed during calm breathing or short apnea phases to minimize the translational movement of the cardiac structures. Incomplete images due to rib shadows or lung overlaps can lead to inaccurate measurements. Another challenge is distinguishing between compacted and non-compacted myocardium [19, 30]. The spaces between the non-compact myocardium can often be better visualized by myocardial contour enhancement with accentuated rendering [30]. The Simpson’s method has initially been defined by vertical biplane planimetry [31]. However, for practical reasons, the mitral annulus in the 2- and 4-ChV was chosen as the basis for echocardiographic LV planimetry [26, 32], resulting in LV planimetry using two planes that differ by only 60°.

All apical views should show the LV apex and the center of the mitral valve in the middle of the scan area to avoid foreshortening. Strict apical alignment can be verified by simultaneous triplane scanning [33]. Image acquisition in the apical views should begin with the aLAX, as foreshortening can be best detected and thus avoided in this view. From the aLAX, clockwise rotation in 60° increments leads to standardized apical 2- and 4-ChV.

The terms normokinesia, hypokinesia, akinesia, and dyskinesia refer to changes in LV wall thickness and not to changes in endocardial contour (inward, absent, outward motion). The endocardium is not smooth but distinctly trabeculated, especially at the LV apex and the inferior-inferolateral and lateral LV segments. Trabeculation leads to a non-compacted myocardium, which should be included into the LV-volumes as part of the LV cavity. According to the recommendations [9], the papillary muscles are also part of the LV cavity and must be included in the LV volume.

LVEF changes of ± 5% between two consecutive examinations cannot be considered a consistent change in LV function [34], and at least three serial examinations showing a consistent change in LVEF are required before a therapeutic decision can be made based on LVEF measurements [35]. It can certainly be helpful to use semi-automatic measurement (Auto-EF) for 2D LVEF assessment [36]. Auto-EF defines myocardial the regions of interest (ROI) using artificial intelligence (AI). It measures the largest and smallest LV areas by speckle tracking the endocardial contour. Manual correction of endocardial contour may be performed if deemed appropriate, but this significantly increases variability and reduces the reproducibility of the measurement results.

Cardiac arrhythmias can affect the accuracy of cardiac volume and LVEF calculations. It is recommended to average three consecutive cycles in sinus rhythm and at least five consecutive cycles in atrial fibrillation (AF) [37, 38]. Since averaging multiple cardiac cycles is time consuming, the use of “index beats” corresponding to an optimal heart rate between 60 and 80/min has emerged as an alternative method. In general, a progressive increase in heart rate is always associated with a corresponding decrease in LVEF.

Determining the maximum and minimum LA volume index (LAVI_max_, LAVI_min_) is currently the preferred method for measuring LA size [39, 40]. LAVI_max_ and LAVI_min_ should be measured using the modified biplane Simpson’s method at the end of LV systole (1–2 frames prior to mitral valve opening) in the apical 2- and 4-ChV, when the LA chamber has reached its largest size and is fully visualized, and should be indexed to body surface area [15, 25]. A useful functional LA parameter is the LA emptying fraction (LAEF), which is calculated from the parameters of maximum and minimum LA volume.

Standardized LV apical views may underestimate LA volume. The LV and LA axis are usually not identical. Therefore, apical images optimized for the LV midline often result in LA foreshortening. This can be avoided by acquiring special LA views, but these often result in LV foreshortening. The LA length should not differ by more than 5 mm between 2 and 4-ChV. When tracing the endocardial border, basal tracing should begin at the mitral valve and cover the entire LA, with the exception of mitral valve tenting area, the origin of the pulmonary veins, and the LA appendage (LAA) [25, 41]. The LA length should be measured from the center of the mitral annulus to the center of the LA superior wall. Although there are several methods for measuring LA volume using 2D echocardiography, the above mentioned is the currently preferred method [15]. If the endocardial LA border cannot be traced correctly in both apical views, the biplane area-length method or a single-plane approach can be used instead. The latter algorithm often yields larger LA volumes than the biplane disc summation algorithm [39].

Key points: Assessment of LV volume should be performed in standardized apical views, which should always be checked by the length of the LV longitudinal axis, which should not differ by more than 5 mm at end-diastole. The boundary between compacted and non-compacted myocardium can only be marked with sufficient certainty if the spaces between the trabeculae of the myocardium are made visible after appropriate image optimization. LA volumes continue to be determined in 2D echocardiography in LA axes that determine the LA cavity in atrial-focused 2- and 4-ChV by biplane planimetry.

### LV- and LA-volume measurements by triplane and 3D echocardiography

Triplane LV volume analyses enable improved determination of LV volume in cases of abnormal LV geometry compared to biplane LV analysis. However, triplane measurements can only be performed if foreshortening is ruled out in all three planes, as otherwise the LV volumes will be underestimated. Speckle tracking in triplane mode requires frame rates of over 40/s. 3D volume measurements—including 3D LVEF and 3D LAEF assessment—may be more accurate, show low variability between studies, and avoid foreshortening if image quality is good. Limitations include lower temporal and spatial resolution compared to 2D echocardiography. Biplane and triplane echocardiography have the potential to improve standardization without sacrificing as much spatial resolution as multidimensional imaging. Furthermore, the reproducibility of automated cardiac 3D volume analyses is generally excellent. However, there are still significant LVEF variations between the different modalities, underscoring the need for consistent longitudinal imaging using the same technique [42].

The quantification potential of 3D is underscored by higher LV volumes and more accurate LV mass assessments [43, 44] (Supplementary Fig. S2). For the acquisition of 3D data sets, the respective target structures—in particular, cardiac volumes and cardiac walls—should be completely captured in the 3Dvolume during diastole and systole [45, 46]. Before acquiring3D volume data, this can be verified using the triplane technique or the so-called multislice technique in short axis views from the apical window. For 3D volume measurement of the left ventricle and left atrium, single beat acquisition with a minimum volume rate of 15/s is preferable to near-real time full-volume data acquisition over 4–6 cardiac cycles in order to avoid stitching artifacts. Stitching artifacts during multi-beat acquisition are generally unacceptable for quantitative assessment of cardiac volumes [47]. Correctly adjusted gain settings are necessary to improve image quality and achieve a high signal-to-noise ratio of the wall structures, thereby minimizing artifacts. Lower volume rates may smooth the actual maximum and minimum volumes too much. Volume rates that are too low in real time may require multi-beat acquisition.

The limitations of echocardiography due to poor image quality can be overcome by using ultrasound contrast agents [48, 49]. Contrast bubbles penetrate the spaces between the trabeculae, allowing good differentiation between compacted and non-compacted myocardium, which often results in larger LVEDV values compared to native echocardiography [50, 51].

Key points: If the endocardial contour of the compacted myocardium is clearly visible, 3D assessment is superior to triplane assessment, which in turn is superior to biplane assessment. The frame rate in triplane mode should be higher than 40/s and the volume rate in 3D mode higher than 12/s.

### LV- and LA-strain measurements

For color Tissue Doppler imaging, users are advised to record 3 cycles with frame rates of more than 100/s. To do this, the image area can be narrowed, and the image depth reduced. The angle between the myocardial walls and the ultrasound beam should ideally not exceed 15°.

Strain derived from speckle tracking has been available since the early 2000 s [52] and offers a user-friendly way to calculate regional and global myocardial wall deformation. Today, AI-based automation allows all three apical views to be processed within seconds without user interaction to obtain an average global longitudinal strain value, while biplane LV volumes and ejection fraction are automatically provided as a by-product of this analysis process [53]. Nevertheless, user monitoring of the tracking process is still recommended.

**Fig. 1:**
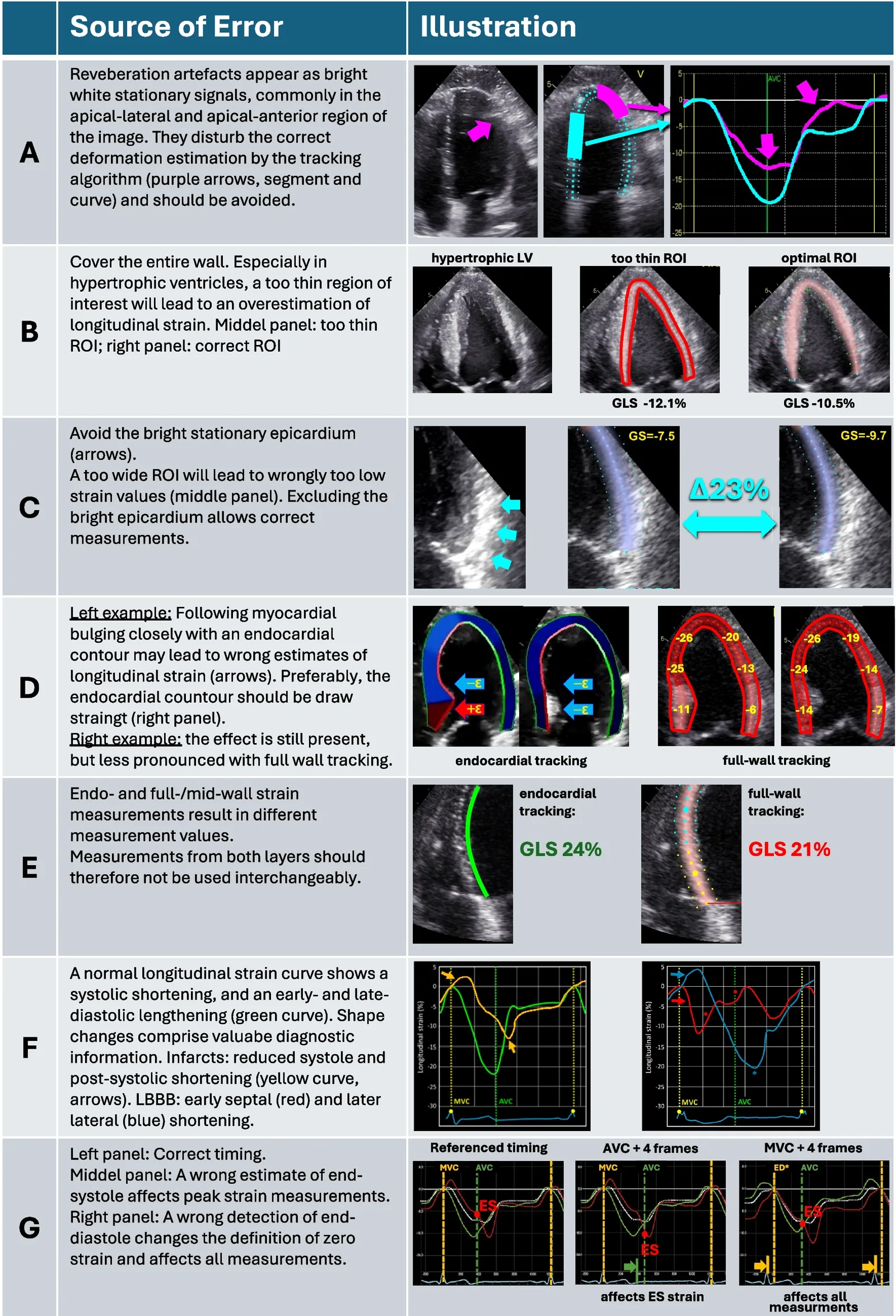
Sources of errors and the illustration of left ventricular strain assessment. Explanation in the figure

Reliable speckle tracking measurement is only possible with good quality images of the complete myocardial wall in all LV segments. Reverberation artifacts, especially in the apical lateral and anterior regions, should be avoided. Another common error that leads to overestimation of regional apical and global strain is foreshortening. Breathing maneuvers can help to reduce this. Echocardiographic images should be stable, free of artifacts caused by probe moving or beathing, and recorded without arrhythmias (Fig. 1A). The ECG signal should be stable and allow reliable QRS triggering. During processing, the ROI should cover the entire myocardium, from the endocardial contour to the subepicardium (Fig. 1B). Care should be taken to avoid the bright and immobile pericardium, as this reduces the measured strain values (Fig. 1C). Papillary muscles should be excluded. Some software solutions only allow endocardial tracking; however, full-wall strain takes more speckles into account and is therefore more robust and, for geometric reasons, less susceptible to foreshortening and regional changes in wall thickness [54].

Strict adherence to the endocardial contour in hearts with septal bulging can lead to incorrect estimates of regional strain and reduced global values, especially with software that only considers the endocardium. To avoid this, the tracking contour should be drawn straight through the bulge (Fig. 1D). This problem is less pronounced with software that performs full wall tracking [55].

The tracking result should be visually verified by comparing the movement of the tracking indicators with the movement of the underlying myocardium.

The two parameters of longitudinal myocardial deformation—full and endocatrdial strain—differ in their values (Fig. 1E). Therefore, they cannot be used interchangeably, and the myocardial layer measured should be specified in the report [56]. Longitudinal strain measurements from different layers do not differ in terms of reproducibility or clinical significance. Consequently, calculating longitudinal strain “gradients” across the wall has no additional diagnostic value, as all layers behave similarly [57]. For the reasons of robustness mentioned above and due to the fact that most available literature references are based on this, the writing committee recommends the use of mid/full-wall strain. Tracking based global strain values show a good reproducibility and are suitable for follow-up examinations [58]. Software solutions from different providers have led to differences in the past. A recent study shows that these differences have become less relevant with modern software versions from the major providers [59]. Segmental strain values exhibit lower reproducibility, and different software solutions vary more in their accuracy in representing regional differences [60, 61]. Therefore, segmental deformation analysis should focus on the shape of the strain curve rather than on the values (Fig. 1F).

Myocardial deformation is a cyclical process, which is why the definition of “zero strain” is arbitrary. The ECG trigger is usually used as substitute for end-diastole as a reference [56]. In patients with conduction delays and correspondingly wide QRS complexes, this ECG trigger may occur too late in the QRS complex and no longer correspond to end-diastole. In this case, Doppler-based mitral valve closure should be used as the time reference instead [62]. Similarly, end systole is often estimated automatically by the software based on the smallest LV volume, and strain values are then reported. These estimates can be incorrect, especially in cases of extensive pathology within an image plane, and the definition of end systole should be corrected based on the Doppler-derived aortic valve closure (Fig. 1G) [62].

Strain derived from speckle tracking was used early on to measure LA deformation [63]. While LV strain tracking algorithms were used initially, companies have recently begun offering software solutions specifically tailored to the LA anatomy. The LA strain values measured with this specialized software do not differ between vendors. Nevertheless, their use significantly improves the reproducibility of measurement and is therefore highly recommended [64].

**Fig. 2:**
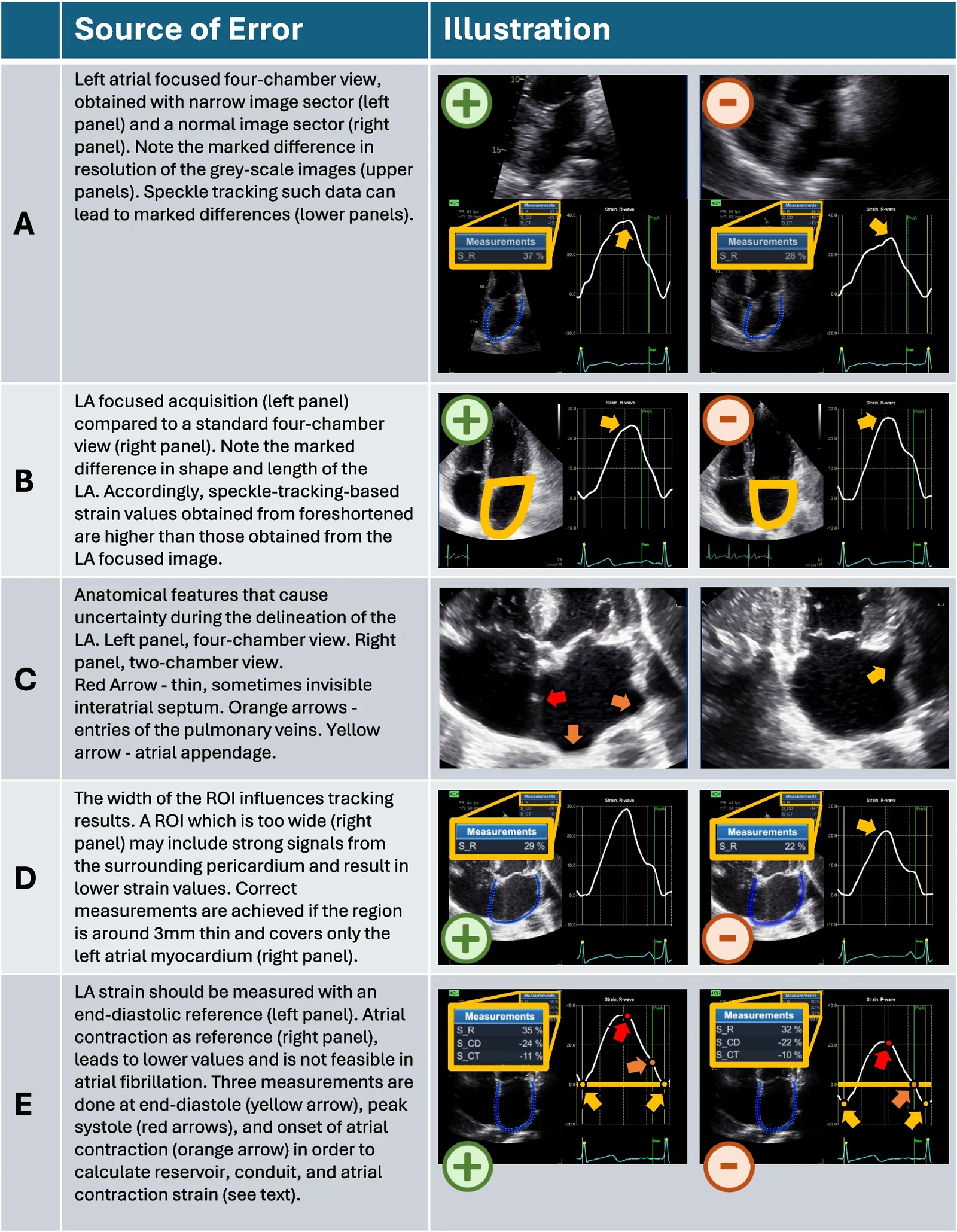
Sources of errors and its illustration of left atrial strain assessment. Explanation in the figure

Typically, LA strain is measured bi-plane in dedicated apical LA-focused 2- and 4-ChV views, and have a narrow sector width to improve image resolution and frame rate (Fig. 2 A and B) [65]. The images should be free of artefacts caused by probe movement or respiration. Care should be taken to ensure that a stable ECG signal is available, which enables reliable triggering on the QRS complex and ideally shows also shows the P wave as a temporal reference. In the case of AF, the third of a group of three consecutive beats with (almost) equal cycle length should be measured. Alternatively, several measurements can be averaged.

Tracking of the LA contour is mainly based on detecting the movement of the myocardial contour. There are hardly any intramyocardial “speckles” that can be tracked. This is due to the poor lateral resolution in the area of the atrial roof, the non-contractile thin tissue of the septum, and the relevant gaps in the contour in the lateral wall due to LAA and pulmonary veins. Currently, it is common practice to track the LA contour by extrapolating these gaps (Fig. 2C). In contrast to the LV wall, the LA wall is thin, and so there is no need to distinguish between endocardial tracking and mid-/full-wall tracking. When using mid-/full-wall tracking software, care must be taken to adjust the ROI to the thickness of the LA wall. Pericardial structures should be avoided as they are immobile and can reduce the measured strain (Fig. 2D) [65].

Longitudinal LA deformation is only measured globally. LA reservoir strain (LASr), which basically reflects the amplitude of the LA strain curve, is commonly reported. LA conduit strain (LAScd) is measured as the difference between the strain at onset of atrial contraction and strain at end-systole. Contraction strain is the difference between end-diastole (zero) and the onset of atrial contraction (LASct) (Fig. 2E) [65]. While the QRS trigger of the ECG is generally used as a reliable substitute for end-diastole, the definition of the onset of LA contraction is less reliable. Some publications define zero strain at the onset of atrial contraction. Both approaches have the same diagnostic potential, however, absolute strain values cannot be directly compared (Fig. 2E) [63].

Key points: To analyze longitudinal LV strain, cine loops with sufficient image quality should be available that show the entire width of the LV wall for complete full wall strain analysis. The longitudinal axis of the LV chamber should be located in the center of the sector in each view. In case of inhomogeneities in LV wall thickness, the respective tracking contour should be drawn straight through the LV wall. For LA strain analysis, the LA wall should always be clearly visible in biplane views throughout the cardiac cycle.

### Automatic LV- and LA-volume measurements

Whereas triplane analyses are performed by manual endocardial contouring, automated or semi-automated analysis is performed in 3D data sets. The automatically detected myocardial contours within the 3D data set can be checked in section planes and manual corrections can be made in post-processing by changing the suggested endocardial contour with mouse clicks. The distinction between compacted and non-compacted myocardium is difficult to recognize, requires sufficiently good 3D images, and must be checked by changing the slice planes within the 3D data set. The delineation of the mitral annulus includes the tenting area in the 3D LV volume analysis. 3D echocardiography improves the accuracy of LV and LA volume assessments by capturing their actual geometry. 3D voxel tracking of the endocardial boundaries allows complete delineation of the endocardial contour without mathematical assumptions, resulting in a “beutel” reconstruction of the left ventricle and left atrium throughout the cardiac cycle. Automated detection is based on good image quality and a minimum acquisition rate of ≥ 12 volumes/s [39, 66]. These measurements suggest that LAEF may be underestimated by 2D echocardiography compared to 3D echocardiography [67].

Automatic volume analyses and LVEF and LAEF assessments combine AI-driven ROI definition with voxel-tracking algorithms. These are based on reference databases that may incorporate underestimations from foreshortened or incorrectly delineated 2D volumes. Automated or semi-automated volume measurements detect and track the LV and LA endocardial boundaries and may minimize errors by manual tracing. AI is increasingly being integrated in the assessment of LV and LA volume and function. The use of AI algorithms can shorten examination time and reduce interobserver variability [36, 68]. AI volume analyses have a high reproducibility, but this also applies to the reproduction of errors. There is ongoing debate as to whether automatically derived stroke volumes are systematically lower than Doppler-derived values, suggesting that the end-diastolic contours may be placed too centrally within the LV cavity [43]. These algorithms optimize clinical workflows by increasing accuracy, robustness, and efficiency [68, 69]. Most vendor solutions allow for easy review and manual adjustment of automatically generated contours prior to final interpretation.

When performing a longitudinal strain analysis, the simultaneous assessment of LVEF is a “by product” that is now provided by several strain software products. It should be noted that such EF values may differ slightly from a dedicated EF analysis using manual or automated measurements, as the optimal definition of a ROI may differ between strain and EF analysis.

Automatic tracking and tracing should be visually compared to the movement of the underlying myocardium and manually corrected, if necessary. Irregular shapes of the LV ventricle and small cavities may require more extensive corrections.

Key points: Currently, automatic detection of the endocardial LV and LA contours should always be visually checked and corrected manually if necessary. The values for LVEDV are often too low in automatic measurements, resulting in too low values of LVSV and LVEF.

### Additional conventional echocardiographic options to assess cardiac function

In the small subgroup of patients who continue to have poor image quality despite all technical measure, dynamic assessment of mitral annular plane systolic excursion (MAPSE) can still provide valuable information as a simple substitute for LVEF [70]. The systolic index of LV contractility (LV dP/dt_max_) calculated from the continuous-wave (CW) Doppler spectrum of mitral regurgitation [71] indicates the rate of pressure increase during the isovolumic contraction phase, but appears to be a less accurate measure of systolic function than LVEF. In addition, the myocardial performance index (MPI or Tei-index) is another parameter for global LV systolic and diastolic performance [72]. The prerequisites for measuring LV dP/dt_max_ using 2D and Doppler echocardiography are the presence of a mitral regurgitation and the correct alignment of the regurgitant jet. In this case, LV dP/dt_max_ reflects the instantaneous pressure difference between the left ventricle and left atrium during systole. MPI is based on time intervals by pulsed wave (PW) Doppler or tissue Doppler echocardiography and combines both systolic and diastolic cardiac performance. The MPI is calculated from the time interval between mitral valve closure and opening and the ejection period between aortic valve opening and closure. The MPI is the ratio of the sum of isovolumetric contraction and relaxation time (determined by the difference of both time intervals) and the ejection time An MPI below 0.40 is considered normal [72]. One of the limitations of MPI assessment is that it is not practical to determine the Tei index in patients with arrhythmias [73].

**Fig. 3:**
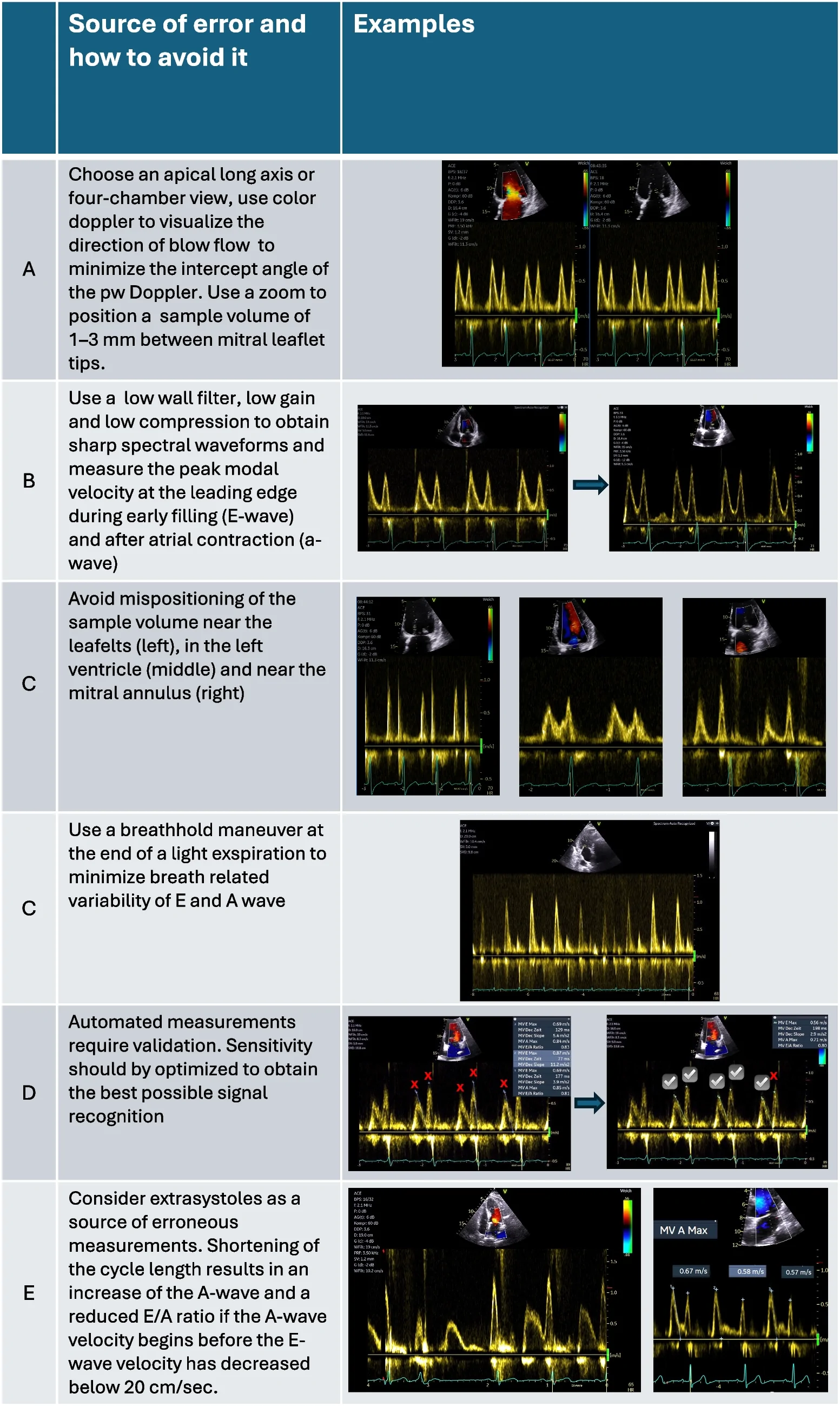

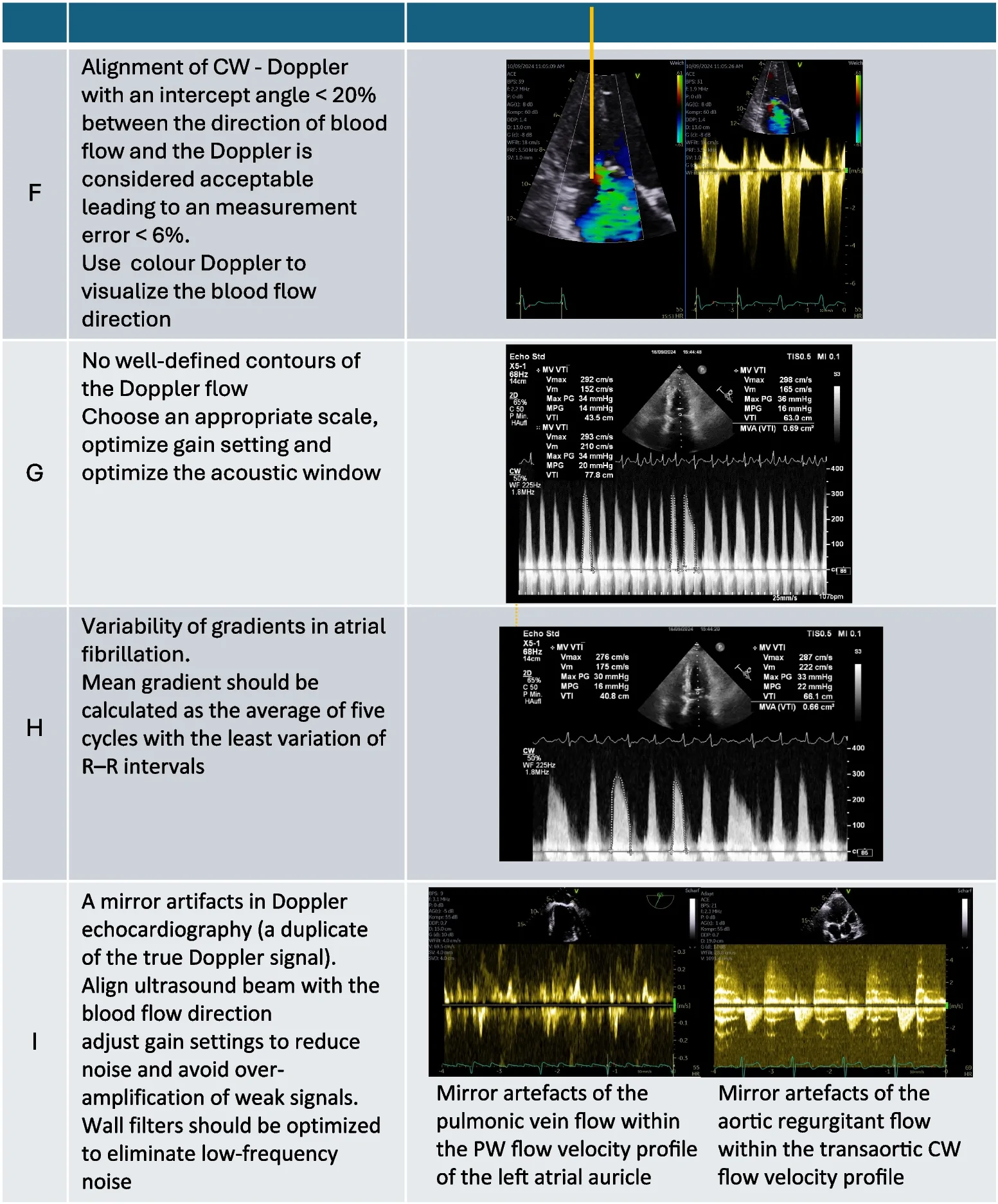
Sources of errors of pulsed wave Doppler assessment determined for transmitral flow and how to avoid them in respective illustrations. Explanantion in the figure

Transmitral PW Doppler recordings form the basis for classifying diastolic LV function [74]. All Doppler spectra require methodological prerequisites in terms of proper standardization and optimization (Fig. 3). The technical procedure for acquiring a PW Doppler spectrum consists first of obtaining a good 2D image, then the best visualizing of the flow phenomena with color Doppler echocardiography, and finally positioning the sample volume precisely at the location where the flow velocity is to be assessed (Supplementary Fig. S3) [6]. To verify the position of the PW sample volume, it is recommended to record the spectra in duplex or triplex mode. With “frozen” 2D cineloops, it is not possible to supervise the correct position of the PW sample volume. Increasing the Doppler frequency results in sharper contours of the spectrum with less penetration. Smaller sample volume sizes can lead to a significant overestimation of maximum velocities [75]. When measuring the PW Doppler spectra, the brightest part within the signal envelope—the so-called modal velocity—should be traced [74]. The modal velocity represents the dominant velocity of blood flow within the sample and is less dependent on gain settings than the outer curve.

For transmitral flow analysis, PW Doppler acquisition is performed in the aLAX or 4-ChV. The sample volume is typically set to a width of 1–3 mm and positioned at the level of mitral valve coaptation between the tips of the mitral leaflets during early diastole. Mispositioning of the PW-Doppler sample volume too far below or above the tips of the mitral valve leaflets results in lower velocities (Fig. 3), while artifacts are recorded if the sample volume is positioned too close to one of the leaflets. Respiratory-related fluctuations in E and A wave can be minimized by taking measurements at the end of expiration (Fig. 3). Automated spectrum detection of E- and A-wave using AI algorithms works best with sharp spectral waveforms without artifacts. The sensitivity of these automated algorithms can be adjusted to achieve the best possible alignment of the automated measurements. The merging E- and A-waves should not be considered for measurements if they are due to ectopic beats or varying PR-time intervals (Fig. 3).

PW Doppler spectra of the LVOT flow allow assessment of forward LV stroke volume (LVES). Positioning of the sample volume within the LVOT is easy in normal LVOT anatomy, as there are almost no differences in diameter between the virtual aortic annulus and the LVOT. In principle, the sample volume in the LVOT should be positioned at the point where the LVOT diameter was determined at mid systole. For this reason, it is logical to acquire the LVOT spectrum in the aLAX, as the position of the sample volume in this view can be compared and verified with the measurement position of the LVOT diameter from the parasternal LAX. In patients with septal bulging, it is recommended to position the PW sample volume closer to the aortic valve anulus [4, 76]. In patients without aortic regurgitation (AR) and/or shunts, the forward LV stroke volume (LVSV) corresponds to the total LVSV (LVSV_tot_). In AR patients, effective LVSV (LVSV_eff_) can be determined by PW Doppler examination of blood flow in the right ventricular outflow tract (RVOT). LVSV_tot_ and LVSV_eff_ determined by PW Doppler echocardiography can serve as a plausibility check for LV volumes and LVSV_tot_ determined by LV planimetry or LV volumetry [19]. Hemodynamic measurements of LVSV_eff_, cardiac output, and cardiac index using Doppler techniques generally yield higher values than planimetry or volumetry (due to foreshortening or inaccurate delineation of endocardial contours) and are comparable to other methods suggesting that forward LVSV can be assessed more reliably using this approach [77, 78].

Transmitral and transaortic CW-Doppler is routinely used to assess pathological flow conditions in valvular heart diseases and cardiomyopathies [1, 79]. Optimized alignment of the CW Doppler beam with an angle below 20° to the direction of blood flow results in an acceptable measurement error of less than 6% (Fig. 3). Transvalvular gradients should be reported together with heart rate, as higher heart rates can lead to an overestimation of mitral stenosis severity [80]. During CW Doppler examination, t the ultrasonic beam should be aligned parallel to the jet so that the maximum velocities can be documented.

### Verification of measurements to avoid errors

There are numerous ways to verify our own echocardiographic measurements. Normal findings are ideal for learning and teaching echocardiographic measurement procedures, as measurements of the same parameters such as LV volumes, LVSV and LVEF, as well as LV wall thickness and LV mass, must provide consistent values across different modalities.

Key points:

1. Diameters in comparable planes, e.g., in the parasternal and apical long axis, be comparable and, due to the different radial and lateral resolution, should only differ by one to two millimeters.
2. The length of the LV longitudinal axes in the apical views should not differ by more than 5 mm. Exceeding this distance leads to LV volumes that are too small and possibly to an LVEF that is too high.
3. Diastolic and systolic LV mass values should not differ by more than 5% from each other; otherwise, the myocardial boundaries will be determined incorrectly.
4. Under normal cardiac conditions, LV stroke volume determined by Doppler echocardiography and planimetry and/or volumetry corresponds to the RVSV, and the total and effective stroke volumes are also equal. Deviations of more than 5% should indicate sources of error that can and should be minimized by qualified work under good acoustic conditions.
5. PW Doppler spectra suitable for evaluation always show the contour of the modal velocity.
6. In pathologies, LVSVtot is always greater than LVSVeff.
7. The effective stroke volume can be reliably determined by Doppler echocardiography above the RVOT if the diameter of a circular reference area or the corresponding cross-sectional area associated with the sample volume has been correctly determined by 3D echocardiography and there is no pulmonary valve regurgitation or left-to-right shunt.
8. In mitral or tricuspid regurgitations, LVSVtot of the respective ventricle should always be greater than LVSVeff. The respective regurgitation volume cannot be greater than LVSVtot, and the difference between LVSVtot and regurgitant volume must still allow for a life-compatible LVSVeff, i.e., usually an effective cardiac output above 4 L/min.
9. In aortic valve regurgitation, LVSVtot corresponds to forward LVSV. LVSVeff corresponds to RVSVeff, which can be determined at the competent pulmonic valve.
10. If the heart valves are intact, the LV forward stroke volume—especially in aortic valve stenosis—can be checked for plausibility using LV planimetry or LV volumetry.

## Summary

This expert proposal describes possible sources of error that can occur in conventional echocardiography when assessing the left heart. Important aspects of ultrasound device settings, standardization, and measurement methodology are discussed for measurements from M-mode, 2D images, and 3D volume data sets. Taking these explanations into account plays an essential role in recording correct, hemodynamically reproducible echocardiographic parameters, which are indispensable for the application of quantitative echocardiography.

Appendix: Conference discussion

Andreas Hagendorff (Leipzig): An important topic at the “Deutscher Echokardiographie Kongress 2024” in Leipzig was the possibility of identifying and avoiding sources of error in echocardiography. Therefore, the present expert proposal deals with the topic of common sources of errors when in the determination of basic echocardiographic parameters. *To start the conference discussion: Do you believe that linear M-mode and 2D parameters are still relevant in clinical scenarios?*

*Bernhard Haring (Homburg/Saar):* In my opinion, LV diameters properly determined by anatomical M-mode or 2D measurements are still important for clinical decision-making. In addition, LV wall thickness is an important parameter. In contrast, volume measurements based on linear parameters are no longer up to date. H*owever, I would like to provocatively ask whether M-mode measurements – especially Feigenbaum`s M-mode sweep—are still state of the art and should peerformed at all?*

*Stephan Stöbe (Leipzig*): Due to its high spatial and temporal resolution, M-mode remains helpful and effective for many issues. In addition, the M-mode sweep is still used in our laboratory as a training tool for testing technical skills and is considered as an echocardiographic “driving” license. *Why do we need to be careful to avoid sources of error when it comes to echocardiographic skills today?*

*Roland Brandt (Bad Nauheim):* There is a modern trend toward performing disease-oriented echocardiography. As a result, aortic valve sclerosis, for example, now only leads to TAVI and mitral valve regurgitation only leads to clipping, without first making a proper diagnosis of the underlying cardiac disease and its severity. It is therefore more than justified to return to the basics of fundamental echocardiography and to bring echocardiography back into focus as a method*. What are the most important methodological aspects in general in this context?*

*Jens-Gerrit Kluge (Göttingen):* The answer is simple: the optimal individual adjustment of the ultrasound settings and clear visualization of the important cardinal structures in standardized imaging. *But back to linear measurements: What should definitely no longer be done?*

*Aydan Ewers (Bochum):* Linear LA measurements from the M-mode and the parasternal LAX are definitely outdated, incorrect, and misleading. In addition, all other linear measurements in non-standardized planes should be avoided. *What phenomenon generally complicates the distance-length measurements and contour delineation of the endocardial contour in a 2D image?*

*Ali Hamadanchi (Jena):* In principle, it often seems as if contours are easy to recognize in the moving image. However, when measurements have to be taken in a still frame from the cineloop, it becomes clear how poor a 2D image can be. For this reason, the best image quality must always be ensured. *For example, how can the LV endocardial contour be defined with the boundary between compacted and non-compacted myocardium?*

*Joscha Kandels (Leipzig):* The spaces between the trabeculae must be enhanced in the image settings. This can be achieved using contour enhancement algorithms (e.g. in GE by maximizing the ACE and hybrid settings). *However, how can modifying the 2D image in this way lead to incorrect image settings?*

*Nicolas Merke (Berlin):* In any case, you must ensure that the gray scale range is not too narrow (compression button). If too few gray scales are set, structures with low echogenicity may be overlooked. *However, is there a way to learn how to delineate the LV endocardial contour in native echocardiography?*

*Andreas Helfen (Lünen):* Contrast echocardiography is very well suited for imaging the LV endocardial contour. After numerous contrast examinations and the comparison of measurements between contrast and native images, it is possible to recognize the boundary between compacted and non-compacted myocardium more intuitively in further native examinations*. However, what is another important source of error in planimetric and volumetric assessments?*

*Dariush Haghi (Ludwigshafen):* When tracing the endocardial contour of the left ventricle in apical views, the height of the mitral valve anulus is often incorrectly placed towards the LV apex, although it should be placed more towards the base of the heart. This leads to truncation of the basal LV layer, resulting in a significant underestimation of LV volumes—especially of LVEDV.* What tips and tricks are there for standardizing intersecting planes in the 2D echocardiography?*

*Ertunc Altioc (Aachen):* If a 3D transducer is available, the biplane technique can be used for parasternal standardization to avoid a secant view of the LV and LA, and the triplane technique optimally to avoid LV and LA foreshortening. *What do errors in determining LV volume and LVEF mean for clinical decisions? Is it even necessary to determine these values correctly?*

*Karsten Lenk (Leipzig):* These values are very important. The classifications of heart failure and the indications for cardiac resynchronization for example, depend exclusively on LVEF, making quantitative echocardiography crucial in this context. In addition, hemodynamic plausibility in the quantification heart valve regurgitation depends largely on correct ventricular volumes. *What other quantitative echocardiographic parameters are also important for quantitative analysis and prognostic statements?*

*Henrik ten Freyhaus (Cologne):* The parameters LAVI_max_ and LAVI_min_, as well as LAEF, are crucial for characterizing diastolic LV function, detecting previously undetected AF and determining the overall prognosis of our patients. *But what do you do if you cannot standardize the 2D image?*

*Christoph Sinning (Hamburg):* Nowadays, this question is easy to answer. With a good acoustic window that provides good 2D images that cannot be standardized, it is currently possible to record a 3D data set from which all standardized cross-sectional images can be reconstructed in post-processing. This allows valid measurements of all cardiac volume parameters as well as LVEF and LAEF to be performed. *Despite this promising possibility, what limitations still exist in 3D echocardiography?*

*Elena Romero-Dorta (Berlin):* The spatial resolution of 3D echocardiography is poorer than that of 2D echocardiography due to technical problems. In addition, the interactions between volume size, spatial resolution, and temporal resolution must be taken into account. The better the spatial resolution, the poorer the temporal resolution. The larger the volume, the poorer the spatial and temporal resolution. *What does this scenario mean for the available volume analysis software?*

*Maximilian von Röder (Leipzig): *A minimum volume rate of approximately 12/s or more is required for the software to analyze cardiac volumes. In addition, there are problems with contour detection in the far field, for example in the left atrium. However, due to the relatively simple geometry of an ovaloid, this volume can be easily to approximate and therefore easily determined in 3D tools. *What tools are available to improve the temporal resolution of 3D data sets?*

*Tarek Bekfani (Magdeburg):* If there are no arrhythmias and the patient is cooperative during the breathing maneuvers, composite 3D volumes from multiple cardiac cycles can be acquired without so-called stitching artifacts. Alternatively, acoustic windows that are less commonly used for cardiac volume analyses, such as the subcostal window, can be used more frequently for 3D volume analyzes if the image quality is good. *A specific question: For which heart valve diseases is 3D TTE most helpful?*

*Jan Knierim (Berlin):* TTE is primarily used for functional analysis of heart valve defects, while transesophageal echocardiography is preferred for morphological assessment. LV volume analysis plays an importantrole in assessing the severity of aortic valve regurgitation, especially when compared to RVSV_tot_and RVSV_eff_. These 3D volume analyses provide a precise method for minimizing misjudgments associated with semi-quantitative parameters. *Does the same apply to mitral valve regurgitation?*

*Tobias Ruf (Mainz):* In any case. Misjudgments of the severity of mitral valve regurgitation are primarily caused by underestimating LV volumes in planimetric measurements and overestimating regurgitation volumes using the 2D PISA method. 3D echocardiography is an essential component of hemodynamic plausibility testing using quantitative echocardiography—but only if it is performed professionally. *Moving on to another topic: Should we now determine other parameters in addition to LVEF to characterize LV function?*

*Ole Breithardt (Kassel):* Of course—this also includes the parameters LVSV_eff_ using Doppler echocardiography and global longitudinal LV strain. Following the introduction of LV strain measurements using tissue Doppler imaging, tracking technology was further developed. Due to the robustness of global LV strain compared to LVEF, global LV strain is currently established for assessing systolic function. *However, what methodological aspects should be considered in strain analysis? What are the obvious strengths of LV speckle tracking especially in clinical use?*

Jens-Uwe Voigt (Leuven): Good image quality is crucial for strain analysis—both in 2D and 3D. An unclear representation of the LV wall, e.g. due to lack of contact between the transducer and the skin or interference from the lungs, leads to incorrect tracking and can result in reduced LV deformation values. The tracking result should always be checked against the underlying myocardial motion. Full wall myocardial tracking should be preferred, and the region of interest should be drawn straight through local thickness changes, i.g. in the case of a localized septal bulge. The biggest advantage of global strain measurements is their sensitivity to subtle functional changes and their reproducibility due to automated analysis. User interaction should therefore be limited to what is absolutely necessary. What additional positive aspects does strain imaging offer?

*Fabian Knebel (Berlin):* LV speckle tracking is used not only to describe systolic LV function, but also to detect LV strain patterns that indicate specific diagnoses. So-called apical ballooning can indicate Takotsubo syndrome and “cherry on the top” patterns can indicate amyloidosis. However, other diseases such as Fabry disease or hypertrophic cardiomyopathy also show typical patterns. *Are there any other limitations for general clinical applicability?*

*Wolfgang Fehske (Cologne):* A current problem of speckle tracking is the inconsistency of software analyses between the different vendors. However, the industry is working to minimize these differences. The major advantage of LV speckle tracking remains its high reproducibility, despite all the limitations and methodological subtleties that still exist. *In which clinical areas can this advantage be demonstrated?*

*Sebastian Kruck (Ludwigsburg):* An undisputed advantage of speckle tracking is the detection of changes in regional LV contractility when comparative examinations are performed out with the same ultrasound device. This means, for example, that induced regional wall motion abnormalities during exercise can be objectively assessed using speckle tracking. *But aren`t, there are other important areas where LV speckle tracking analysis can help?*

*Raluca-Ileana Mincu (Essen):* According to current recommendations, oncology patients must be monitorerd very closely using echocardiography, which poses a major challenge. If these patients undergoing chemotherapy could be examined quickly and objectively with reproducible results by the same examiner using the same device, this would be an excellent way to solve this problem. At the same time, the problem of questionable comparability between different examiners and vendoprs highlights the current limits of AI in echocardiography. *Can we identify the strengths and weaknesses of the commercially available AI algorithms?*

*Sebastian Ewen (Villingen-Schwenningen):* The AI algorithms used in echocardiography are currently based on verified image data sets. These obviously still have limitations—for example effective LV volumes are very often measured too small—and lead to some implausible results. They must therefore be continuously reviewed and presumably replaced in part by better validated data sets. Methods using AI that are obviously incorrect, such as quantifying mitral valve regurgitation based on the size of jet area, should not be established. The strength of current AI algorithms lies in the extreme reproducibility and speed of analyses, whichsimplify the clinical workflow. *What is the main conclusion of our expert proposal?*

*Andreas Hagendorff (Leipzig):* All statements in the current expert proposal—not least in our discussion—point to the importance of methodological knowledge in echocardiography in order to make the right diagnoses and not overlook important ones. This means that more space should be created in training, during internships, in courses, but also at national and international conferences to address and discuss such issues. Turning on an ultrasound machine without using the image optimizatoion buttans and taking into account the physical laws of ultrasound should not become the norm in our daily clinical practice. We must all ensure that subtle methodological knowledge is not lost to users in echocardiography.

## Supplementary Information

Below is the link to the electronic supplementary material.

ESM 1 Supplementary Figure S1: Illustration of M-Mode measurements of the left ventricular (LV) myocardium. In A an anatomical M-Mode is presented in standardized manner in a patient with hypertensive heart disease. In B error sources and differences of linear measurements in the LV setpum are illustrated by using inner-edge-to-inner-edge or leading-edge-to-leading edge method with and without including the moderator band, in C in the posterior wall with and without including trabecula. The proper values are labeled in red. (PDF 375 KB)

ESM 2 Supplementary Figure S2: Illustration of 2D measurements the left ventricular (LV) myocardium in A. Illustration of LV volume measurements in B as the basis for 3D LV mass assessment in C. (PDF 458 KB)

ESM 3 Supplementary Figure S3: Illustration of the influence of sample volume position on pulsed wave Doppler spectra in the left ventricular outflow tract (LVOT) and left ventricular (LV) cavity. In A the 2D apical long axis view is presented, in B the corresponding midsystolic color-coded Doppler to obtain an aliasing zone in the LVOT that runs almost parallel to the aortic valve plane to minimize angular error. In C and D the sample volume is too far in the LV cavity (orange circle). The positions of the red in E and F is the proper one, if LVOT diameter is determined at the respective positions (red circle). In G and E the sample volume is too distal to the AV (yellow circle), in I and J the sample volume ist o near tot he anterior mitral leaflet (blue circle). (PPTX 2.07 MB)

## Data Availability

The data used in this manuscript are available.
