# Supplementary material for: How can common errors in left heart echocardiography be avoided? An expert proposalby the German Working Group of Cardiovascular Ultrasound
Source: Clin Res Cardiol. 2025 Oct 27;115(12):2085–104. doi: 10.1007/s00392-025-02780-8 (PMC13751758; doi:10.1007/s00392-025-02780-8)
Supplement: Supplementary file 1 [file 392_2025_2780_MOESM1_ESM.pdf]

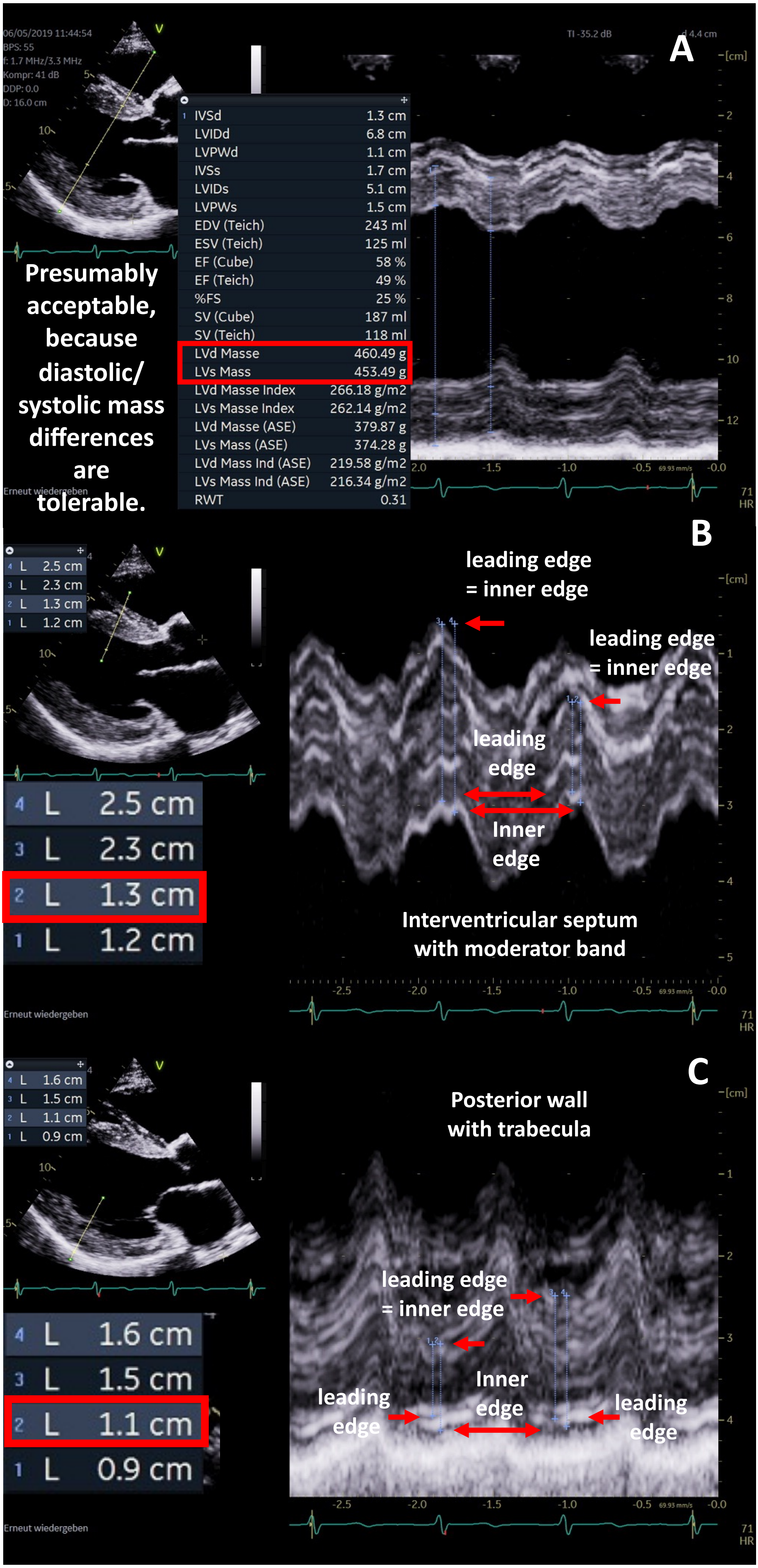
A
06/05/2019 11:44:54
BPS: 55
f: 1.7 MHz/3.3 MHz
Kompr: 41 dB
DDP: 0.0
D: 16.0 cm
TI -35.2 dB
d 4.4 cm
IVSd 1.3 cm
LVIDd 6.8 cm
LVPWd 1.1 cm
IVSs 1.7 cm
LVIDs 5.1 cm
LVPWs 1.5 cm
EDV (Teich) 243 ml
ESV (Teich) 125 ml
EF (Cube) 58 %
EF (Teich) 49 %
%FS 25 %
SV (Cube) 187 ml
SV (Teich) 118 ml
LVd Masse 460.49 g
LVs Mass 453.49 g
LVd Masse Index 266.18 g/m2
LVs Masse Index 262.14 g/m2
LVd Masse (ASE) 379.87 g
LVs Mass (ASE) 374.28 g
LVd Mass Ind (ASE) 219.58 g/m2
LVs Mass Ind (ASE) 216.34 g/m2
RWT 0.31
Presumably acceptable, because diastolic/ systolic mass differences are tolerable.
Erneut wiedergeben
71 HR
B
4 L 2.5 cm
3 L 2.3 cm
2 L 1.3 cm
1 L 1.2 cm
leading edge = inner edge
leading edge = inner edge
leading edge
Inner edge
Interventricular septum with moderator band
Erneut wiedergeben
71 HR
C
4 L 1.6 cm
3 L 1.5 cm
2 L 1.1 cm
1 L 0.9 cm
Posterior wall with trabecula
leading edge = inner edge
Inner edge
leading edge
leading edge
Erneut wiedergeben
71 HR
