# Supplementary material for: How can common errors in left heart echocardiography be avoided? An expert proposalby the German Working Group of Cardiovascular Ultrasound
Source: Clin Res Cardiol. 2025 Oct 27;115(12):2085–104. doi: 10.1007/s00392-025-02780-8 (PMC13751758; doi:10.1007/s00392-025-02780-8)
Supplement: Supplementary file 2 [file 392_2025_2780_MOESM2_ESM.pdf]

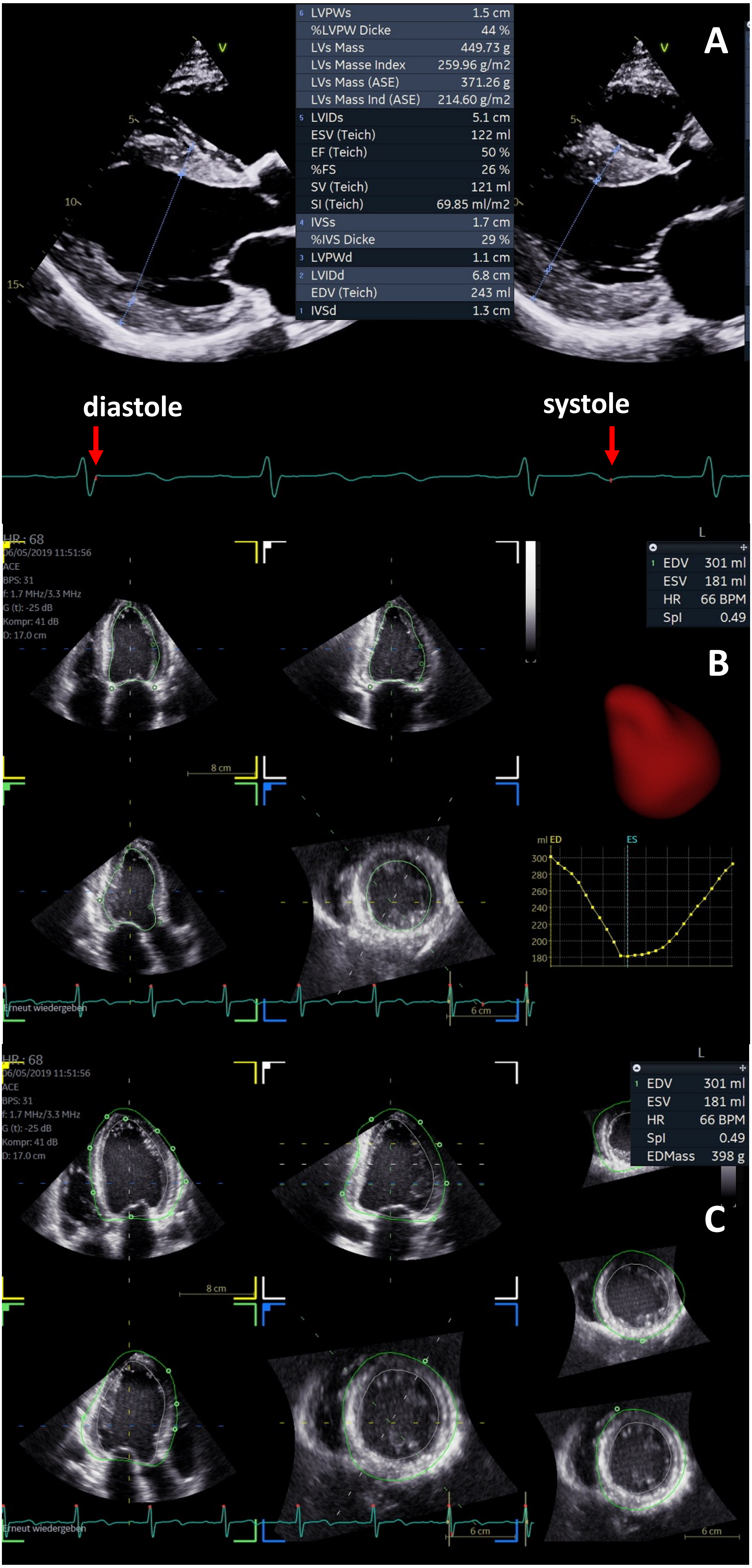
A
LVPWs 1.5 cm
%LVPW Dicke 44 %
LVs Mass 449.73 g
LVs Masse Index 259.96 g/m2
LVs Mass (ASE) 371.26 g
LVs Mass Ind (ASE) 214.60 g/m2
LVIDs 5.1 cm
ESV (Teich) 122 ml
EF (Teich) 50 %
%FS 26 %
SV (Teich) 121 ml
SI (Teich) 69.85 ml/m2
IVSs 1.7 cm
%IVS Dicke 29 %
LVPWd 1.1 cm
LVIDd 6.8 cm
EDV (Teich) 243 ml
IVSd 1.3 cm
diastole
systole
B
HR: 68
06/05/2019 11:51:56
EDV 301 ml
ESV 181 ml
HR 66 BPM
SpI 0.49
ml ED
ES
300
280
260
240
220
200
180
8 cm
6 cm
Erneut wiedergeben
C
HR: 68
06/05/2019 11:51:56
EDV 301 ml
ESV 181 ml
HR 66 BPM
SpI 0.49
EDMass 398 g
8 cm
6 cm
Erneut wiedergeben
